# Brain mechanisms of auditory perception in autism spectrum disorder: a comparative perspective on tonal and non-tonal languages

**DOI:** 10.3389/fnsys.2026.1628536

**Published:** 2026-05-12

**Authors:** Anastasia Neklyudova, Yangyang Liu, Yiyun Xia, Olga Sysoeva

**Affiliations:** 1 Laboratory of Human Higher Nervous Activity, Institute of Higher Nervous Activity and Neurophysiology, Russian Academy of Science, Moscow, Russia; 2 Suzhou Research Institute of Shandong University, Suzhou, Jiangsu, China; 3 Nanjing Normal University of Special Education, Nanjing, Jiangsu, China; 4 Sirius Center for Cognitive Sciences, Sirius University of Science and Technology, Sochi, Russia

**Keywords:** auditory cortex, auditory perception development, autistic spectrum disorder, pitch processing, tonal and non-tonal languages

## 1 Introduction

When we perceive complex sounds, such as speech or music, our auditory perception systems need to process multiple sound features and integrate distinct characteristics across different timescales. Recent research has highlighted neurophysiological mechanisms that underlie auditory perception, such as neural synchronization with the temporal structure of external stimuli and rate coding across different frequencies, including those corresponding to the fundamental frequency (F0) of complex sounds (Nourski and Brugge, 2011; Wang, 2018). Synchronization mechanisms appear particularly important for the encoding of fast changing phonemic information, while rate coding contributes to prosody perception which detects sound variations over longer time windows (Giraud and Poeppel, 2012; Rosen, 1992). These mechanisms may be altered in neurodevelopmental conditions such as autism spectrum disorder (ASD). Children with ASD often exhibit atypical auditory processing (O'Connor, 2012), which may contribute to their difficulties in speech perception and language acquisition (Mody and Belliveau, 2013).

The vast majority of existing studies have focused on speakers of non-tonal languages, particularly English, thereby overlooking populations of children with ASD who grow up speaking tonal languages, where pitch carries lexical meaning and auditory processing demands may differ. This opinion paper aims to synthesize recent findings on auditory perception, examine cross-linguistic differences at neurophysiological level, and explore perspectives for studying these processes in populations with ASD. We propose that electroencephalographic (EEG) markers hold significant promise for investigating how auditory perception varies across language backgrounds in individuals with ASD. Given its high temporal resolution, EEG-based biomarkers are particularly well-suited for capturing both synchronized and rate-coded neural responses. In addition, EEG is non-invasive and cost-effective, making it ideal for use in pediatric and clinical populations.

## 2 Brain mechanisms of auditory perception

Pure tone perception relies on the tonotopic organization of the cochlear basilar membrane and the phase-locking properties of auditory nerves, which tend to generate spikes at specific phases of acoustic waves (Oxenham, 2018). However, timing information becomes increasingly less precise at higher stages of the auditory processing pathways. For example, in the inferior colliculus of the midbrain, phase-locked responses are detectable at and below 1,000 Hz (Liu et al., 2006), while in the auditory cortex, the threshold is about 100 Hz (Lu and Wang, 2000; Nourski and Brugge, 2011). As temporal precision decreases along the auditory pathway, cortical processing must rely on different mechanisms for encoding sound information. At the cortical stage of auditory processing, different types of neurons support auditory perception: stimulus-synchronized spiking neurons and non-synchronized neurons that rely on rate coding, meaning that they are selectively tuned to specific fundamental frequency (F0; Nourski and Brugge, 2011; Wang, 2018). Thus, the analysis of complex auditory stimuli, including speech stimuli, may be the results of coordinated involvement of these different mechanisms. By complex auditory stimuli, we refer to sounds that contain multiple spectrotemporal features that must be integrated over time, such as speech and music.

Stimulus-synchronized activity in humans is often studied presenting amplitude-modulated tones or click trains. Such stimulation elicits an evoked EEG response aligned with the frequency and phase of external stimulus and reflects the precision of synchronization between neuronal and auditory signals across trials which can be estimated as inter-trial phase coherence, or ITPC (Stroganova et al., 2020). This response has the highest amplitude when stimuli vary with the frequency of 20–40 Hz (Picton et al., 2003). Rhythmic stimuli varying within this range are perceived as roughness—a perceptual quality intermediate between distinct pulses and sounds with pitch. This response is associated with gap detection and in older adults, with speech in noise perception (Dheerendra et al., 2021; Ross and Fujioka, 2016). It is also associated with rate discrimination of rhythmic stimuli varying at frequency around 27 Hz in children (Neklyudova et al., 2026). Moreover, cortical oscillations at low-gamma frequency (25–40 Hz) are associated with phoneme perception (Giraud and Poeppel, 2012; Leong and Goswami, 2014). Taken together, these findings suggest that rhythmic responses in this frequency range may play an important role in processes that rely on precise temporal cues.

A rate coding mechanism can be detected with EEG when periodic stimuli that convey a discernible pitch (F0) are introduced. Pitch perception typically emerges around 40 Hz and becomes more salient at higher frequencies (Krumbholz et al., 2000). Recent studies associated pitch processing with a sustained wave (SW), a brain response which is elicited by continuous periodic sounds (click trains or vowels) and persists until the stimulus ends (Gutschalk et al., 2002; Keceli et al., 2012; Orekhova et al., 2024). During the presentation of rhythmic click trains, SW amplitude increases with the frequency of stimulation (Gutschalk et al., 2002; Keceli et al., 2015). Notably, the involvement of the SW in discriminating rhythmic stimuli around 40 Hz has also been demonstrated in children (Neklyudova et al., 2026). Overall, the converging evidence supports the view that the SW represents a rate-dependent neural mechanism that is preferentially sensitive to higher repetition frequencies at which the stimulus gives rise to pitch perception rather than merely distinct pulses or roughness.

These two brain responses also have different developmental trajectories: while ITPC increases with age, reaching its peak during adolescence (Cho et al., 2015), SW either remains stable or, according to some reports, even decreases with age (Arutiunian et al., 2022; Neklyudova et al., 2024; Stroganova et al., 2020). Thus, SW may reflect neural mechanisms particularly important in early development for shaping auditory perception. Unfortunately, SW is much less studied both in non-tonal and tonal languages. An interesting, yet unexplored, research question is whether this response plays a greater role in speech development in tonal compared to non-tonal languages.

## 3 Language experience and brain mechanisms of pitch perception

Pitch is a fundamental feature of all languages. Both in tonal and non-tonal languages pitch variation is crucial for the perception of prosody, including features such as intonation and stress. At the same time, in tonal languages such as Mandarin Chinese, Vietnamese, or Thai, pitch modulations, known as lexical tones, serve not only prosodic but also lexical functions, distinguishing word meanings. Importantly, lexical tone perception relies on pitch contour variations over relatively short time windows, typically at the syllable level (hundreds of milliseconds), whereas intonation and stress perception involve tracking pitch changes over longer durations, spanning words or entire sentences.

Evidence indicates that experience with a tonal language influences pitch perception. Specifically, tones tend to be perceived in a more categorical manner, leading to better discrimination across category boundaries compared to equivalently separated stimuli within the same category. Studies have shown that speakers of tonal languages have narrower boundary widths between tones (Morett, 2020; Peng et al., 2010). This sensitivity could be linked to enhanced neural plasticity in pitch processing regions, such as the superior temporal gyrus (STG; Bhaya-Grossman and Chang, 2022). Meta-analysis has shown that only tonal language speakers consistently recruited the left STG for lexical tone processing—an area implicated in phoneme processing in non-tonal languages (Liang and Du, 2018). At the same time, variation in intonation on sentence level elicits bilateral or rightwarded activation in both tonal and non-tonal language speakers (Gandour et al., 2003).

One of the theories describing the functional asymmetry in the auditory cortex is the asymmetric sampling in time hypothesis, according to which, the left auditory cortex processes auditory information that varies in time windows of 25–80 ms, whereas the right auditory cortex operates over timescales of 150–250 ms (Oderbolz et al., 2025; Poeppel, 2003). This division of labor appears to be rooted in differences in architectonic structure, connectivity patterns, and the heterogeneity of temporal receptive fields at the level of single neurons across the two hemispheres (Buxhoeveden et al., 2001; Caeyenberghs and Leemans, 2014; Cavanagh et al., 2020). Syllables in Mandarin Chinese, the most frequently studied tonal language, have an average duration of approximately 250 ms (Peng, 2006), which exceeds the temporal window of 25–80 Hz that is considered optimal for left-hemisphere processing. Therefore, it remains unclear which specific mechanisms in the left hemisphere contribute to the processing of lexical tones, and how this sensitivity to lexical tones is shaped in such languages. To address this knowledge gap, future research should examine how pitch variations are integrated across different temporal windows in speakers of both tonal and non-tonal languages. This apparent contradiction also highlights that findings on hemispheric specialization based on non-tonal languages may not be fully generalizable to tonal language contexts. Thus, there is a pressing need for more cross-linguistic studies to better understand the neural basis of auditory perception in diverse language systems.

**Figure 1:**
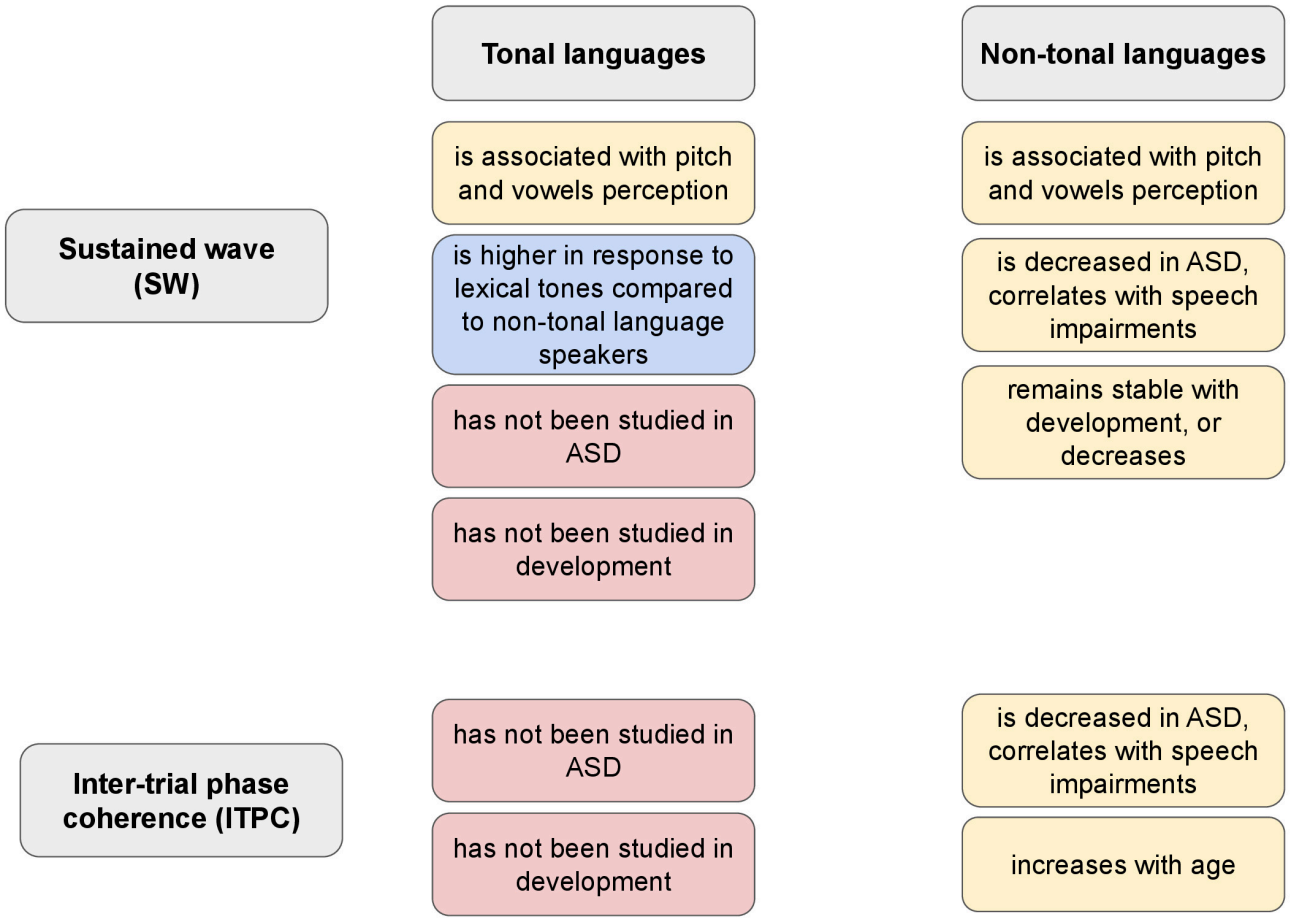
Summary of recent findings on sustained wave (SW) and intertrial-phase coherence (ITPC) in ASD research and cross-linguistic studies. The blue box highlights the differences between tonal and non-tonal languages, and the red boxes highlight the current gap in knowledge.

The described above EEG responses of synchronized and non-synchronized activity can be a well-suited tool for studying these processes from the perspective of auditory development and its impairment across different language environments. Of particular relevance to our opinion paper is the finding that SW has been reported to be enhanced in speakers of tonal languages compared to those of non-tonal languages, but only when semantically meaningful syllables (with tones) were presented (Fan et al., 2017). Similar results were also found for auditory brainstem frequency-following response (FFR). Speakers of tonal languages show stronger FFR than English listeners (Krishnan et al., 2010). However, this result characterizes only adults, and not neonates (Jeng et al., 2011), suggesting that tone language experience sharpened neuronal sensitivity to linguistic pitch information at the auditory brainstem level (Figure 1).

## 4 Altered brain mechanisms of auditory perception in ASD

Disruptions in either of the described mechanisms (synchronized activity and rate-coding activity) can significantly affect the development of speech perception. This is particularly relevant in neurodevelopmental conditions such as ASD, where atypical atypical auditory processing in early infancy may trigger cascading effects on speech development and broader cognitive functions. Indeed, children with ASD exhibit difficulties in the development of both auditory and speech perception (Mody and Belliveau, 2013; O'Connor, 2012).

Several studies have reported reduced ITPC response in individuals with ASD (De Stefano et al., 2019; Seymour et al., 2020), although findings remain inconsistent (Edgar et al., 2016; Stroganova et al., 2020). Also, some studies have found that this response is associated with speech difficulties in children with ASD (Arutiunian et al., 2023; Roberts et al., 2021). All these studies were conducted on speakers of non-tonal languages.

SW is also impaired in ASD (Arutiunian et al., 2023; Fadeev et al., 2024; Stroganova et al., 2020). In the non-tonal language context (Russian), SW correlates with the ability to perceive words in noise in children with ASD (Fadeev et al., 2024). It is possible that the impaired pitch-processing mechanism has an even more significant effect in children with ASD from tonal language contexts, since this response has higher amplitude in individuals with tonal language background (Fan et al., 2017). Studies have shown that children with ASD from tonal language backgrounds exhibit deficits in pitch processing, but only in the speech domain, while their discrimination between pure tones or melodic contours are even enhanced (Jiang et al., 2015).

Taken together, these findings highlight the language-specific role of pitch processing in ASD and support the utility of neurophysiological markers for tracking atypical speech perception mechanisms.

## 5 Discussion

In this opinion paper, we reviewed studies on the brain mechanisms underlying auditory processing, with a focus on how these mechanisms may differ between speakers of tonal and non-tonal languages. Neurophysiological evidence suggests that the perception of pitch variation engages distinct neural processes depending on linguistic experience. However, it remains unknown how phase- and frequency-synchronized mechanisms, such as ITPC, function in speakers of tonal languages, as this phenomenon has not yet been systematically investigated in this population.

In individuals with ASD from non-tonal language context, both ITPC and pitch-related response (SW) mechanisms appear to be affected (De Stefano et al., 2019; Fadeev et al., 2024; Orekhova et al., 2024). However, these auditory responses have not yet been examined in individuals with ASD who speak tonal languages. Nevertheless, existing evidence indicates that children with ASD with tonal language experience exhibit wider categorical boundary widths compared to typically developing peers, showing reduced categorical perception of lexical pitch (Chen et al., 2022), which might affect language abilities in this population. Similarly, in languages where syllable duration carries lexical meaning (i.e., Finnish or Japanese) categorical perception of duration is also impaired in individuals with ASD (Kasai et al., 2005; Lepistö et al., 2005), whereas this deficit is not observed in languages where syllable duration is not lexically relevant (Huang et al., 2018).

Several limitations of the reviewed studies should be acknowledged. First, substantial variability in stimulation paradigms across studies, together with relatively small sample sizes in some cases, limits direct comparability and generalizability of findings. Importantly, this variability extends to the nature of the auditory stimuli: studies differ in their use of simple vs. complex sounds, synthetic vs. natural speech, listening conditions, and key acoustic properties (e.g., duration, modulation rate, and spectral content). These differences can engage partially distinct processing mechanisms and timescales, complicating the interpretation of inconsistent findings across studies. Notably, studies reporting altered categorical perception of linguistic tones in children with tonal language backgrounds (Morett, 2020; Peng et al., 2010) have predominantly used lexical or pure tone stimuli, which limits the ability to generalize findings to more naturalistic listening conditions. To better understand the neural mechanisms underlying these effects in ASD, future research should incorporate a broader range of stimuli that vary in complexity and ecological validity, allowing clearer dissociation of which aspects of auditory processing are affected. In addition, the marked heterogeneity of ASD may contribute to inconsistent results; however, these challenges can be addressed in future research through larger, well-characterized samples and the use of harmonized experimental protocols. While the present review considers both clinical and cultural differences, maintaining consistency within a given experimental paradigm is critical, and future research should prioritize more standardized stimulus designs

Taken together, these findings underscore the importance of cross-linguistic studies in children with ASD to identify which specific alterations in auditory perception contribute to the development of speech perception deficits within different language environments. It is possible that a common underlying auditory processing mechanism is affected in ASD, but the behavioral and neurophysiological manifestations vary depending on the linguistic context. Tonal languages, which place a high functional load on pitch, offer a valuable model for disentangling language-specific and universal components of auditory processing atypicalities in ASD.
